# Massively parallel experimental interrogation of natural variants in ancient signaling pathways reveals both purifying selection and local adaptation

**DOI:** 10.1101/2024.10.30.621178

**Published:** 2024-10-31

**Authors:** José Aguilar-Rodríguez, Jean Vila, Shi-An A. Chen, Manuel Razo-Mejia, Olivia Ghosh, Hunter B. Fraser, Dan F. Jarosz, Dmitri A. Petrov

**Affiliations:** 1 Department of Biology, Stanford University, Stanford, CA, USA; 2 Department of Chemical and Systems Biology, Stanford University School of Medicine, Stanford, CA, USA; 3 Present address: Altos Labs, Bay Area Institute of Science, Redwood City, CA, USA; 4 Department of Physics, Stanford University, Stanford, CA; 5 Department of Developmental Biology, Stanford University School of Medicine, Stanford, CA; 6 Chan Zuckerberg Biohub, San Francisco, CA 94158, USA

## Abstract

The nature of standing genetic variation remains a central debate in population genetics, with differing perspectives on whether common variants are mostly neutral or have functional effects. We address this question by directly mapping the fitness effects of over 9,000 natural variants in the Ras/PKA and TOR/Sch9 pathways—key regulators of cell proliferation in eukaryotes—across four conditions in *Saccharomyces cerevisiae*. While many variants are neutral in our assay, on the order of 3,500 exhibited significant fitness effects. These non-neutral variants tend to be missense and affect conserved, more densely packed, and less solvent-exposed protein regions. They are also typically younger, occur at lower frequencies, and more often found in heterozygous states, suggesting they are subject to purifying selection. A substantial fraction of non-neutral variants showing strong fitness effects in our experiments, however, is present at high frequencies in the population. These variants show signs of local adaptation as they tend to be found specifically in domesticated strains adapted to human-made environments. Our findings support the view that while common variants are often neutral, a significant proportion have adaptive functional consequences and are driven into the population by local positive selection. This study highlights the potential to explore the functional effects of natural genetic variation on a genome scale with quantitative fitness measurements in the laboratory, bridging the gap between population genetics and functional genomics to understand evolutionary dynamics in the wild.

## Introduction

One of the longstanding debates in population genetics centers on the nature of standing genetic variation (1–3). Two contrasting perspectives exist regarding the fitness consequences of genetic variants found at high frequency in natural populations. Classical theory (4), with the neutral or nearly-neutral theory as the latest incarnations (5–9), posits that low-frequency variants often are deleterious and thus are functional, whereas common variants tend to be neutral or nearly neutral and thus are likely to have limited or no functional impact. This perspective commonly serves as the null model for studies of genetic variation in the wild (10). In contrast, balance theory suggests that a substantial proportion of common genetic variation is functionally important, beneficial in at least a subset of environments, and maintained at high frequencies through the action of balancing selection (11). Balancing selection can operate through several mechanisms, including overdominance (heterozygote advantage) (12), frequency-dependent selection (13), and selection that varies across space (local adaptation) (14) or time (fluctuating selection) (15).

Despite much effort and voluminous data, the current population genetic evidence remains inconclusive about which theory best describes population genetic variation. There is clear evidence for most genes that functional mutations, for instance, nonsynonymous variants—those that alter the amino acid sequence of proteins—tend to occur at lower allele frequencies than synonymous variants (16–22). This implies that they are likely to be disproportionately deleterious. While this pattern is consistent with nearly neutral theory, in the absence of experimental functional data it is hard to know whether the common nonsynonymous or other functional variants are entirely neutral as a class or whether they do contain a substantial fraction of balanced, selectively consequential variants, and debates regarding the adaptive value of standing genetic variation remain unresolved. Moreover, quantitative genetics approaches, such as quantitative trait loci (QTL) mapping or genome-wide association studies (GWAS), almost universally lack the spatial resolution necessary to determine the fitness consequences of single nucleotide variants, which are precisely the type of genetic polymorphism most commonly found in natural populations.

Recent advances in precision genome editing now make addressing this question experimentally tractable. Although older CRISPR editing methods suffered from low efficiencies (5–20%), limiting the feasibility of massively parallel editing, new techniques in the budding yeast *Saccharomyces cerevisiae* have substantially improved precision genome editing efficiencies (80–100%) (23–28). This breakthrough now enables the simultaneous introduction of tens of thousands of variants in parallel and the accurate measurement of their fitness *en masse* using genomic barcodes.

**Figure 1.**
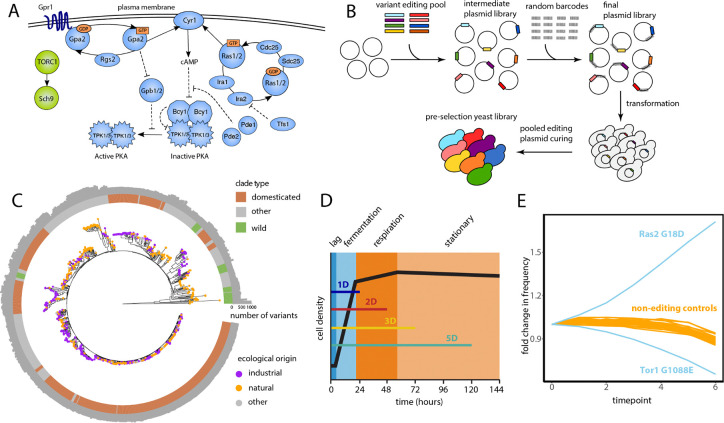
CRISPEY fitness screen of natural genetic variants in nutrient-sensing pathways. (A) The signaling pathways Ras/PKA (blue) and TOR/Sch9 (green) in yeast. Positive signaling interactions are depicted by full lines and negative interaction by dashed lines. (B) Schematic of the CRISPEY-BAR+ approach: from cloning to pre-selection yeast population. The association between random barcodes and editing oligos is obtained by sequencing the final plasmid library. (C) Maximum likelihood tree of the 1,011 yeast strains. Clades are labeled according to Tengölics *et al.* (45) into domesticated, wild, or other. Individual strains are labeled according to their ecological origin based on the provenances reported by Peter *et al.* (17) into industrial, natural, or other. We report the number of variants in the Ras/PKA and TOR/Sch9 pathways for each strain. (D) Schematic of the four experimental conditions with varying time before transfer (1D: 24 h, 2D: 48 h, 3D: 72 h, 5D: 120 h) to change the amount of time spent in different phases of the growth cycle (lag, fermentation, respiration, and stationary). (E) Example of competition data over time from the 2D condition. Each blue line represents the average across three experimental replicates of the normalized counts for a beneficial (Ras2 G18D) and a negative missense edit (Tor1 G1088E). The beneficial variant is oncogenic in humans (*RAS2* is an ortholog of *KRAS*) (32). Counts in later time points are normalized to the first time point. All the barcodes mapping into the same edit are counted. Orange lines represent the normalized counts for barcodes associated with non-editing oligos and represent a neutral fitness baseline.

In this study we have employed one such method, CRISPEY (24), to map with high precision the fitness effects of approximately 10,000 natural genetic variants across four environments in the core Ras/PKA and TOR/Sch9 pathways, which control cell proliferation in response to environmental stimuli across all eukaryotes (29–31) (Fig. 1A). These pathways are crucial for the virulence of several pathogenic yeasts, and the pathogenesis of diverse human cancers (32), and are often mutated to adaptive states in experimental evolution (33–38). Both standing genetic variation and *de novo* mutations within them contribute to the adaptation to novel nutrient niches in yeast (33–42), indicating that they may act as a “hot-spot” of evolutionary novelty and possibly functional genetic variation segregating in this species. The variants we investigate encompass the majority of the known coding and non-coding genetic variation in these pathways across the species (17).

Here we confirm that large-effect functional variants tend to be disproportionately deleterious but also surprisingly often present at high frequencies in the population. Moreover, we find that these high-frequency variants are enriched in domesticated and industrial strains, suggesting that they have been driven to high frequency by local positive selection. These results provide strong support in favor of the balance theory of natural variation. This study not only highlights the potential of high-throughput precision genome editing to resolve foundational questions in population genetics, but also expands the field of empirical fitness landscapes beyond single-gene approaches, by probing entire biological pathways at a large scale for both coding and regulatory variants, thereby advancing our understanding of the evolutionary dynamics of genetic variation.

## Results

### High-throughput measurement of the fitness effects of natural genetic variation in Ras/PKA and TOR/Sch9 signaling

To quantify the fitness effects of individual genetic variants in the Ras/PKA and TOR/Sch9 pathways we employed a modified version of CRISPEY that differs slightly from previously published versions of the method (24, 27, 28), which we term CRISPEY-BAR+. CRISPEY is a method that generates a precise genome edit per cell, and in a pooled assay, measures their fitness effects by tracking an associated barcode in a plasmid (24). An improvement of the original method, CRISPEY-BAR, is based on a new vector that incorporates two consecutive retron-guide cassettes, allowing the simultaneous generation of two guide/donor pairs for making two precise edits in the same cell (27, 28). One guide/donor pair integrates a genomic barcode in a neutral locus and the other introduces a precise edit of interest elsewhere in the genome. With CRISPEY-BAR+ we also generate one precise edit and a unique barcode per cell and, in a pooled assay, measure the change in abundance of the edited strain through high-throughput sequencing of the chromosomal region where barcodes are integrated (Fig. 1B). However, in contrast to CRISPEY-BAR (27, 28), every guide/donor pair, and thus the variants that they introduce, is associated with hundreds of unique barcodes providing robust internal controls to ensure changes in fitness are linked to genome editing and improving the precision of measurement. We obtained the association between edits and barcodes by sequencing the plasmid library.

After curating the relevant literature, we selected 18 genes from the Ras/PKA pathway and 6 from the TOR/Sch9 pathway (Supplementary Table S1), focusing on those most central to these signaling pathways (Fig. 1A). Using genome-wide polymorphisms identified from sequencing 1,011 genetically diverse yeast isolates from multiples ecological niches (spanning wild, clinical, and domesticated strains among others) (17), we identified 14,384 variants segregating in *S. cerevisiae* within these pathways, of which 9,715 were in open reading frames and 4,669 in neighboring noncoding regions, and including both single-nucleotide variants as well as indels (Fig. 1C). We were able to target with CRISPEY-BAR+ 11,058 of those 14,384 variants, which were all the variants near a PAM site–a requirement for precision genome editing. Across these edits, we observed an average editing frequency of ~85% determined from randomly picked barcoded strains, in agreement with previous studies using the CRISPEY editing technology (27, 28).

To investigate fitness in an environment linked to the function of the Ras/PKA and TOR/Sch9 pathways, we competed the edited pool in a minimal medium with limiting glucose (1.5%, M3) over ~25 generations in serial batch culture (43). We conducted growth competitions under four distinct experimental conditions, each with three technical replicates. The experimental conditions varied based on the duration the cells spent in the flask before transfer: 24 hours (1D), 48 hours (2D), 72 hours (3D), and 120 hours (5D) (Fig. 1D). As the duration of the growth cycle lengthens, the time cells spend in the stationary phase—when they have depleted available nutrients—increases. Previous work with adaptive mutants in the RAS/PKA and TOR pathways in experiments of laboratory evolution demonstrated that these growth conditions should allow us to reveal fitness effects of functional variation in these pathways (38, 42).

During the growth competitions, we sampled cultures every ~4.3 generations and used high-throughput sequencing of the barcode region in these samples to monitor the relative abundance of variants over time (Fig. 1E). We then used these frequency trajectories to estimate fitness effects for each variant. To control for the distribution of neutral fitness measurements that arise in wild-type cells from experimental noise and genetic drift, we measured variant fitness effects from each competition relative to non-editing (random) barcodes that do not alter the genome apart from the barcode insertion (Fig. 1E). We used a Bayesian approach to quantify the selection coefficients for each variant (44). This approach is based on a hierarchical model that employs all experimental replicates simultaneously for the inference of fitness (Methods). We estimated selection coefficients for 9,494 variants. The remaining 1,564 variants did not have barcodes in the competition data.

We detected 3,673 (39%) variants that had significant non-neutral fitness in at least one condition, with an average of 219 beneficial and 1,003 deleterious variants per condition. For these variants, the 95% posterior credible interval for the estimated selection coefficient did not include zero, indicating a high posterior probability that the selection coefficient differs from zero. We found that the fitness effects measured by CRISPEY-BAR+ were reproducible. Fitness estimates from growth competition replicates were similar for non-neutral variants (average Spearman’s ρ across conditions and pairwise replicate comparisons is 0.87, *p*-value < 2.2 × 10^−16^).

The data are also consistent with prior expectations of functionality. Specifically, nonsynonymous variants within this group of non-neutral variants tend to exhibit greater absolute fitness effects than synonymous variants (Wilcoxon’s test, *p* = 5.3 × 10^−7^). There are 984 variants that are non-neutral in at least two conditions. We focus on this set of non-neutral variants, as our results remain consistent with an increasing number of environments in which a variant is considered non-neutral. We reasoned that this choice minimizes the impact of false positives while maintaining a sufficient sample size to ensure statistical power.

The majority of natural variants are neutral in our experimental conditions (61%). While there may be false negatives among these variants, the high overall editing rate in our screen makes it likely that most of them are indeed neutral within the limits of our detection power. This result follows the expectation of neutral theory, especially for core eukaryotic pathways that are highly conserved from yeast to humans, and therefore are expected to evolve under high constraint. Nonetheless, it is remarkable that even within these highly conserved pathways, nearly 40% of variants exhibit detectable functional consequences in at least one of the few environments we examined.

### Non-neutral variants tend to be missense and affect important protein properties

**Figure 2.**
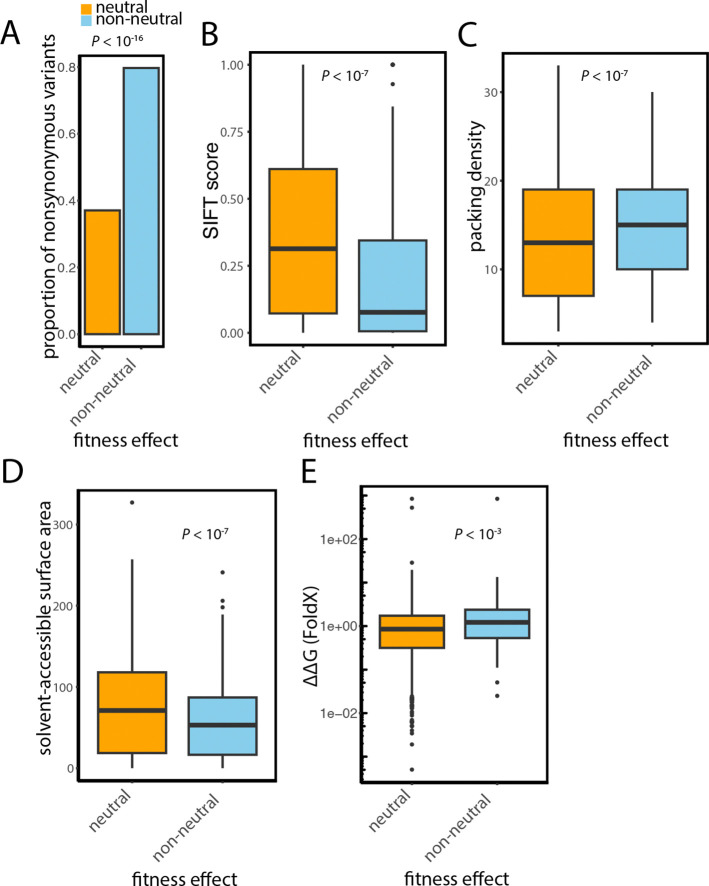
Non-neutral variants tend to be missense and affect multiple protein properties. (A) Bar plot showing the enrichment of nonsynonymous variants among non-neutral variants compared to neutral variants. (B) Box plot showing that non-neutral variants tend to affect more conserved residues (SIFT score). (C) Box plot showing that non-neutral variants tend to affect residues with higher packing density. (D) Boxplot of solvent accessibility for non-neutral and neutral residues. (E) Box plot of protein stability for non-neutral and neutral variants. Boxplots show the median and upper and lower quartiles; whiskers show 1.5 times the interquartile range.

We next sought to identify which types of variants were most likely to have significant effects on fitness, leveraging the single-nucleotide resolution of the measurements. We found a substantial enrichment for missense variants among non-neutral variants (Fisher’s exact test, odds ratio = 2.1, *p* < 2.2 × 10^−16^) (Fig. 2A). We then proceeded to evaluate how these nonsynonymous variants were affecting different protein properties. We first found that if a missense variant occurs within a conserved protein region it is more likely to have an impact on fitness. For this analysis, we used Sorting Intolerant from Tolerant (SIFT) scores that predict whether an amino acid substitution is likely to affect protein function based on sequence conservation and the physical properties of the amino acids involved (46, 47). This score ranges from 0 to 1 and represents the normalized probability of finding an amino acid in a given position in sequence alignments. We find that non-neutral nonsynonymous variants tend to have lower SIFT scores than neutral nonsynonymous variants suggesting they are more likely to affect protein function (Wilcoxon’s test, *p* = 1.2 × 10^−8^) (Fig. 2B). Variants with a SIFT score below 0.05 are considered to have a strong functional impact (46). There is an enrichment of such variants among non-neutral variants in our assays (Fisher’s exact test, *p* = 4.2 × 10^−8^).

We sought next to determine how these non-neutral missense variants impact protein structure by examining their effects on two key structural properties: packing density and solvent accessibility. Packing density reflects how closely a residue is situated within the protein’s tertiary structure, essentially measuring the degree of contact a residue has with other residues. Solvent accessibility represents the surface area of a residue that is exposed to water. Substituting residues in densely packed regions, as well as those with lower solvent exposure, is likely to induce considerable changes to the protein’s structure. The analysis of site-specific protein evolutionary rates has shown that they tend to be correlated with different structural properties but the two main ones are the packing density and solvent accessibility (48).

Residues in more packed regions of a protein, as well as residues less exposed to the solvent, tend to evolve slowly, since changing them is likely to affect protein structure drastically. Here we find that these two protein properties are also important for predicting fitness in our assay. We find that nonsynonymous non-neutral variants tend to affect residues with higher packing density (Wilcoxon’s test, *p* = 1.5 × 10^−8^) (Fig. 2C) and those that are less solvent-exposed (Wilcoxon’s test, *p* = 2.6 × 10^−8^) (Fig. 2D). In contrast, no significant differences in packing density or solvent accessibility were observed between non-neutral and neutral synonymous variants. These results show that non-neutral nonsynonymous variants have a greater overall impact on protein structure than neutral variants as expected.

If an amino acid substitution causes too much strain on the protein structure, it will often destabilize it, resulting in loss of function. FoldX computes the difference in the free energy of unfolding the protein structure before and after a nonsynonymous variant (ΔΔ*G*) (49). Non-neutral variants tend to have higher ΔΔ*G* values compared to neutral variants, indicating a greater impact on protein stability (Wilcoxon’s test, *p* = 3.3 × 10^−4^) (Fig. 2E). If the ΔΔ*G* is high, often above 2, a variant is usually deemed destabilizing. We find an enrichment of such destabilizing variants among non-neutral variants (Fisher’s exact test, *p* = 1.9 × 10^−3^).

In summary, our findings indicate that non-neutral variants are more likely to be missense mutations, affecting conserved, densely packed, and buried residues within a protein, and often resulting in destabilization. These results provide a strong biochemical basis for the distinction between neutral and non-neutral variants, further validating the results of our high-throughput fitness screen.

### Non-neutral variants show signs of purifying selection

**Figure 3.**
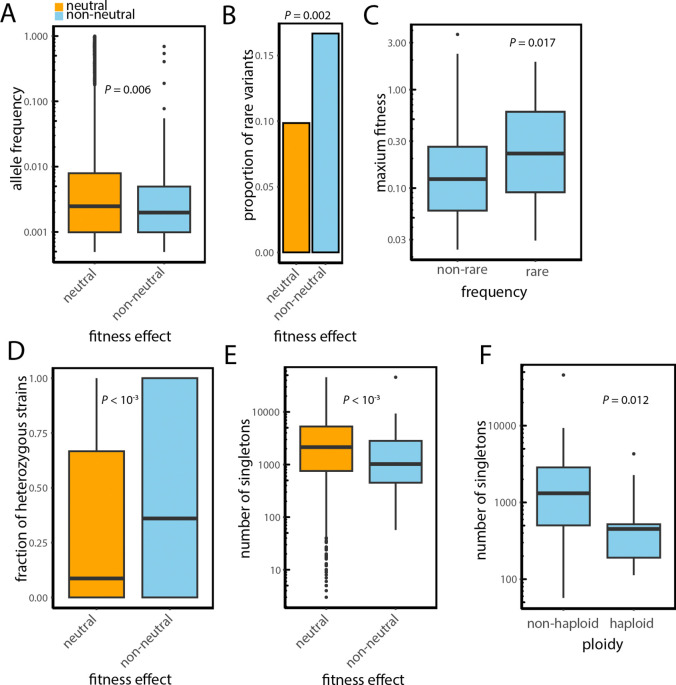
Non-neutral variants show evidence of negative selection. (A) Boxplot showing that non-neutral variants tend to have lower allele frequency. (B) The proportion of rare variants defined as those with the lowest allele frequency is greater among non-neutral variants. (C) Boxplot showing that the experimental fitness impact of rare positive variants is greater than for less rare positive variants. (D) Boxplot showing that the fraction of strains where the variant is on heterozygosis is higher for non-neutral variants. (E) Boxplot showing that singleton non-neutral variants tend to be younger. (F) Boxplot showing that non-neutral singletons in haploid strains tend to be younger. Boxplots show the median and upper and lower quartiles; whiskers show 1.5 times the interquartile range.

The population frequency data is key to test the selective effects of the variants in the population. Consequently, we investigated the frequency of these variants across 1,011 strains of *S. cerevisiae* spanning the ecological diversity of this species. We found that non-neutral variants tend to have lower allele frequency compared to neutral variants (Wilcoxon’s test, *p* = 5.5 × 10^−3^) (Fig. 3A). Similarly, the number of strains where a given variant is present is also smaller for non-neutral variants (Wilcoxon’s test, *p* = 0.028). The proportion of rare variants, defined as those with the lowest possible allele frequency in the 1,011 yeast strains panel (present in just one strain), is also greater among non-neutral variants (Fisher’s exact test, *p* = 0.002) (Fig. 3B). We also find that rarer non-neutral variants tend to have a larger measured fitness impact than more common non-neutral variants (Wilcoxon’s test, *p* = 0.017) (Fig. 3C). Non-neutral variants were also more likely to be heterozygous compared to neutral variants (Wilcoxon’s test, *p* = 3.8 × 10^−4^) (Fig. 3D), further suggesting that these variants are often deleterious, with their detrimental effects being compensated by the presence of dominant wild-type alleles. All of these analyses are consistent with functional variants being held at lower frequency in the population likely due to their deleterious effects as suggested by the classical, nearly-neutral theory.

Beyond studying allele frequency, it is also possible to assess the age of the singleton variants, present in only one strain, with the expectation that more deleterious variants should be younger. To estimate the age of these singleton-carrying strains, we consider the length of the terminal branch by assessing the total number of singletons they possess (50). Younger strains, that is strains that have a closer relative in the phylogeny, typically have fewer singletons, whereas older strains that separated from the rest of the strains in the tree farther back in time, tend to have accumulated more of these variants over time. We find that non-neutral variants indeed tend to be found in younger strains, with nearly 1,500 fewer singleton variants on average (6,014 vs. 7,490; Wilcoxon’s test, *p* = 1.3 × 10^−4^) (Fig. 3E). This finding suggests that these variants are more recent and have not yet been purged by natural selection. Additionally, we find that singleton non-neutral variants in haploid strains tend to be younger compared to those in non-haploid strains, which have around four times more singletons on average (1,491 vs 6,543; Wilcoxon’s test, *p* = 0.012) (Fig. 3F). The phenotypic effects of variants in haploid strains are directly exposed, as there is no second allele at the same site to mask their impact, unlike in diploid strains. Consequently, purifying selection is more effective in removing non-neutral deleterious variants in haploid strains in the absence of the buffering effects for diploidy.

Finally, we tested the prediction that multiple deleterious variants in the same strain should be removed by natural selection at a higher rate and thus we should find less co-occurrence of singleton non-neutral variants in the same strain. This is exactly what we find. The singleton non-neutral variants tend to co-occur in the same strain more than one and half times as frequently as neutral singletons (bootstrap, *p* = 0.018) (Fig. S1A). Note that this pattern is not observed for non-singleton variants that are expected to be less deleterious individually (bootstrap, *p* = 0.078) (Fig. S1B).

Overall, we show that non-neutral variants tend to be rare, occur at lower allele frequencies, are more often found in heterozygosity, and are generally younger than neutral variants. Taken together with the results from the previous section, these observations indicate that the variants that show fitness effects in our laboratory assay are enriched for the variants that are often deleterious in natural populations and are maintained at low frequency under mutation-selection balance.

### Common non-neutral variants show evidence of local adaptation

**Figure 4.**
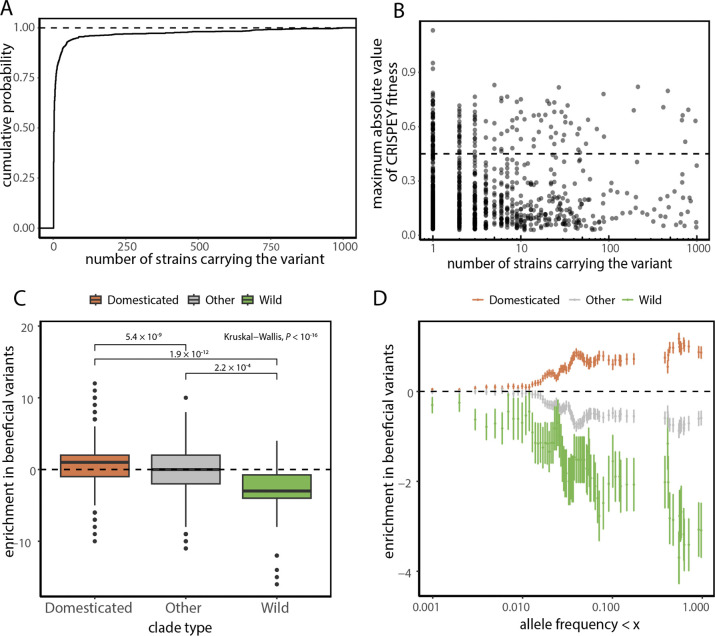
Common non-neutral variants show evidence of local adaptation. (A) The empirical cumulative probability distribution for the number of strains carrying variants that are non-neutral in at least two conditions. (B) Scatter plot displaying the maximum absolute fitness values across the four conditions for variants that are non-neutral variants in at least two conditions, plotted against the number of strains in the 1,011 yeast strain collection carrying the variant. The dashed line indicates a fitness threshold set at twice the average value (0.46). (C) Boxplot showing the difference in enrichment in variants that are beneficial in at least one environment between domesticated, wild, and other clades. The *p*-values for the group comparisons were calculated using the Wilcoxon’s test. Boxplots show the median and upper and lower quartiles; whiskers show 1.5 times the interquartile range. (D) The enrichment in beneficial variants as a function of allele frequency for domesticated, wild, and other clades.

We next sought to investigate variants that show high fitness values despite having a high allele frequency as these are variants whose presence is not expected under the natural expectations of neutral or nearly-neutral theory. Of the 984 variants that are non-neutral in at least two environments, 223 or 22.3% occur in more than 10 strains (Fig. 4A). Among these common non-neutral variants, 26.3% exhibit an absolute fitness value that is more than twice the average across all measurements (Fig. 4B).

It is also instructive to ask the inverse question. Namely what is the proportion of functional variants among all common polymorphisms? The fraction of non-neutrals in at least two conditions among all variants present in more than 10 strains is 9.8%, and 14.1% for nonsynonymous variants. Of these, 18% and 9% respectively, have absolute fitness effects stronger than twice the average. This shows that a substantial fraction of common polymorphism is functionally impactful, and at times strongly so. The totality of observations suggest that while most of the functional polymorphisms are rare in the population, a substantial fraction of functionally consequential polymorphisms are common and, also, that the inverse is also true–a surprisingly substantial proportion of common polymorphisms are (strongly) functional.

These variants could be maintained in the population at high frequency by balancing selection, so we aimed to explore if non-neutral variants are more commonly found in specific clades or associated with specific ecological niches. We initially focused on 17 genetically-isolated clades previously studied by Tengölics *et al.* (45). These authors found clear metabolic differences between strains from the 7 domesticated and 10 wild clades. Budding yeast is the dominant species in the fermentation of various beverages and foods and has been domesticated on several independent occasions. This process of domestication has had a large impact on the stress tolerance and the fermentative growth capacity of this species, which are traits regulated by the core nutrient-sensing pathways we study here. Therefore, we reasoned that some of the natural variants in these pathways may have played a role in the adaptation to human-made environments (domestication).

Of the 535 variants which are non-synonymous and non-neutral in at least two environments a substantial fraction (19.3%) is present in more than 10 strains (Fig. 4B). Notably, 66% of these common non-neutral and non-synonymous variants are exclusively found in domesticated clades whereas only 2.9% are exclusively found in wild clades. To assess enrichment, we compared non-neutral variants against a set of neutral variants with comparable allele frequencies (Fig. S1C,D). These matched controls ensure that our observations are not confounded by differences in the allele frequency distribution between non-neutral and neutral variants or by the phylogenetic structure.

We indeed find that strains in the domesticated clades contain more non-neutral variants than matched control variants whereas strains in the wild clades contain fewer non-neutral variants than matched controls (Wilcoxon’s test, *p* = 5.2 × 10^−4^). The effect is especially striking for non-neutral variants that are beneficial in at least one environment (Wilcoxon’s test, *p* = 1.9 × 10^−12^) (Fig. 4C). We found that this difference in the enrichment of beneficial variants between wild and domesticated clades tends to decrease as more common variants are excluded (Fig. 4D). In other words, common non-neutral variants are enriched specifically in strains from clades adapted to human-made environments.

Some domesticated *S. cerevisiae* clades include strains from natural niches, so we examined whether industrial strains within these clades show a higher enrichment of beneficial variants compared to natural strains, and indeed they do (Wilcoxon’s test, *p* = 8.8 × 10^−4^) (Fig. S2A). Industrial strains in clades that are not classified as domesticated or wild by Tengölics *et al.* (“Other” in Figs. 4C,D) also have a higher enrichment in beneficial variants than natural strains (Wilcoxon’s test, *p* = 1.2 × 10^−4^). We applied a linear mixed-effects model to quantify the impact of a strain’s ecological origin (industrial or natural) across all *S. cerevisiae* clades, treating clade identity as a random effect (Methods; Fig. S3). This analysis revealed that industrial strains harbor a higher number of beneficial variants when controlling for clade identity as a possible confounding factor (effect size = 0.9, *P* = 9.9 × 10^−5^, Fig. S3). So again, considering the ecological origin of strains within clades we find that strains adapted to human-made environments (industrial strains) tend to be more enriched for beneficial functional variants in the RAS/PKA and TOR/Sch9 pathways compared to strains from natural sources, and this effect is also driven by more common variants.

## Discussion

This study provides a comprehensive interrogation of the fitness consequences of natural genetic variation within the Ras/PKA and TOR/Sch9 pathways in *S. cerevisiae*, which are ancient and key regulators of cell proliferation across all eukaryotes. Leveraging our high-throughput CRISPEY-BAR+ approach, we precisely measured the fitness effects for over 9,000 natural variants across multiple conditions, providing insights into the evolutionary forces shaping these core pathways. The scale and precision of this experimental undertaking allowed us to tackle a central and longstanding question in population genetics: the fitness consequences of common variants in natural populations. Our findings challenge the key assumption of neutral theory that most common variants are neutral and instead reveal significant evidence for positive selection driving common variants into the population due to local adaptation to human-made environments.

However, some of our findings do agree with the expectations from neutral theory. Namely, a substantial proportion of natural variants show no significant fitness effects under our experimental conditions. Further, the variants that show fitness effects in our experiments show signs of negative selection, as they tend to be rare and young, and are often found in heterozygous states, which allows the buffering of their detrimental effects on fitness, and tend not to co-occur with each other. Most of the non-neutral variants are also missense mutations affecting densely packed, and buried residues within proteins–features crucial for maintaining their structural integrity. These observations align well with the idea that most standing variation in these highly conserved pathways is under purifying selection, preventing the accumulation of deleterious mutations and keeping the ones that occur at low frequencies.

Interestingly, however, we also observed a substantial set of common (present in more than 10 strains and often much higher frequency) non-neutral variants with high fitness in at least one of the environments we explored, which do not follow the neutral expectation. These variants are enriched in domesticated clades and strains associated with human-made environments, suggesting they may have contributed to local adaptation to these specialized ecological niches. The process of domestication has significantly influenced the stress tolerance and fermentative growth capacity of *S. cerevisiae*, and these are traits controlled by the Ras/PKA and TOR/Sch9 pathways, which aligns well with the idea that positive selection has affected variation in these nutrient-sensing pathways. Another reason for the strong signal of local adaptation we uncover may be that the relatively stable and controlled environments experienced by domesticated yeast strains offer less ecological complexity compared to wild environments, where mutations that lock signaling pathways into specific functional configurations could be highly disadvantageous. The Ras/PKA and TOR/Sch9 pathways are also key targets of adaptation in laboratory experiments, suggesting they may have unique characteristics not shared by other signaling pathways that makes them particularly prone to accumulating local beneficial mutations. Applying our approach to additional pathways could reveal whether these systems influence adaptation in distinct ways.

Our results thus agree more strongly with balance theory than with neutral theory. While most variants in these pathways are neutral or subject to purifying selection, we find a substantial fraction of common variation under positive selection. With the advent of methods for fitness mapping *en masse* at single-nucleotide resolution, like ours, we can begin to disentangle and quantify, for the first time, the relative contributions of neutral evolution and balancing selection in shaping standing genetic variation.

Despite the strength of our findings, this study has several limitations. First, we conducted our fitness assays in a haploid background, while many yeast strains are naturally diploid. Fitness effects in diploid strains could differ for variants with recessive or partially dominant effects. Second, we focused on four environmental conditions, and exploring a broader range of conditions could reveal additional insights into the pleiotropic effects of these variants. Finally, by measuring the fitness of individual variants in a single genetic background, we could not investigate the potential role of epistasis in modulating variant fitness across different genetic contexts.

Future studies using our method could address these limitations by measuring fitness in more strains, including diploid strains, under a wider range of environmental conditions, including more complex media that mimic natural ecological conditions. However, despite all the complexities that we did not explore in this study, one of its more surprising outcomes is the strong correlation between fitness effects measured in the laboratory and ecological adaptations observed in nature. Our work demonstrates that laboratory-based fitness assays can reliably predict real-world evolutionary outcomes, revealing an emergent simplicity that experimental approaches like ours are capable of capturing (51).

Our study also opens the door for applying deep learning approaches to genotype-to-fitness prediction at scales above genes (52). Experimentally, we now have genome-wide, high-throughput precision genome editing tools such as CRISPEY-BAR+ (23–28) that can engineer hundreds of thousands of mutations into the genome and then measure their fitness effects in parallel with high precision across multiple environments. Analytically, the development of deep learning algorithms, such as convolutional neural networks, is revolutionizing biology (53). Such deep learning approaches are not always practical because they require truly large amounts of data. However, the number of individual variants we can now engineer, and study, is of the appropriate scale to allow such approaches to achieve tremendous prediction accuracy. The fruits of such a research program would be more accurate mechanistic and predictive models of how genomic information, at nucleotide resolution, give rise to phenotypes and fitness. Such models could not only advance our understanding of how genetic variation influences phenotypes but also provide powerful tools for predicting evolution.

In conclusion, this study bridges the gap between population genetics and functional genomics by providing direct measurements of fitness effects for natural variants across entire pathways. The comprehensive fitness landscape of natural variation we mapped demonstrates how our massively parallel fitness mapping method can be used to address long-standing questions in evolutionary biology. Future studies that expand this approach to additional pathways and environments and genetic backgrounds will further our understanding of the genotype-fitness map, providing a more nuanced view of the evolutionary dynamics that shape natural populations.

## Methods

### Media

We performed competition experiments in M3 media (43). This media is glucose-limited (1.5% glucose), meaning that the cells run out of glucose before any other nutrient. This media has been widely used in experimental evolution before (54, 38, 42, 55–59). We used SD -histidine -uracil 2% raffinose media for pre-culturing before induction: 6.7 g/L Yeast Nitrogen Base (YNB) (RPI), 1.92 g/L dropout mix synthetic minus histidine, uracil, adenine rich without YNB (US Biological), and 20 g/L raffinose (Sigma). We used SD -histidine -uracil 2% galactose media for induction: 6.7 g/L YNB, 1.92 g/L dropout mix synthetic minus histidine, uracil, adenine rich without YNB, and 20 g/L galactose (Sigma). We also used SC media: 1.7 g/L YNB; 5 g/L ammonium sulfate (ACROS organics), 1.9 g dropout synthetic mix complete without nitrogen base (US Biological), and 20 g/L glucose (Sigma).

### Variant selection and editing library design

We selected natural genetic variants from the 1,011 yeast genomes project (17). We selected 24 genes: 18 from the Ras/PKA pathway and 6 from the TOR/Sch9 pathway (Supplementary Table S1). For each gene, we selected variants located within the coding region as well as those within 500 base pairs upstream and downstream of the coding region, therefore including not only coding variants but also noncoding variants that could potentially impact regulation and gene expression. We designed CRISPEY oligos to edit these variants in the ZRS111 strain, which contains the S288c reference alleles. The library contains 19,906 guide/donor oligonucleotides targeting 11,058 genetic differences. We designed the guides and donor sequences in the oligos as described previously (24). We also included 50 pairs of oligos targeting essential genes. In each pair, one oligo introduces a nonsense mutation to a cysteine or a tyrosine codon, while the other oligo introduces a synonymous mutation in the same codon. Lastly, we designed 30 guides and donors with random sequences which therefore are non-editing (Fig. 1E).

### Library cloning

We ordered an oligonucleotide library from Twist Biosciences. We amplified the oligonucleotide library using primers #310 and #313 with Q5 hot-start DNA polymerase (New England Biolabs). All the primer sequences used in this study can be found in Chen *et al.* (27). This PCR reaction produced amplicons with flanking 20 bp sequences homologous to plasmid pSAC119. We cloned the PCR-amplified double-stranded DNA into NotI-digested pSAC119 using NEBuilder HiFi DNA Assembly Cloning Kit (New England Biolabs). We performed the Gibson assembly with a molar ratio of vector:insert of 1:5.

We transformed the assembled plasmid libraries via electroporation into Endura electrocompetent cells (Lucigen). We performed five electroporations, each with 25 μL of Endura cells and 80 ng of library plasmid DNA. We conducted the electroporations in 0.1cm-gap cuvettes in a GenePulser (Bio-Rad). We allowed the transformed bacteria to recover in recovery media (supplied with Endura competent cells) for one hour, and then plated on LB/Carbenicillin plates for incubation at 37°C overnight. We also plated dilutions to estimate the efficiency of the transformation (cfu/transformation).

We extracted plasmids using the EZNA Plasmid Maxi Kit (Omega Biotek). We eluted the plasmids in 3mL of kit-provided elution buffer. We subsequently digested the samples with NotI and CIP (New England Biolabs) in order to linearize empty vectors. We then ethanol precipitated the plasmids to concentrate the libraries at approximately 0.8 ug/μL.

The base yeast strain ZRS111 has been described previously (24). We performed the transformation into yeast via electroporation of the plasmid library into the strain ZRS111. We prepared electrocompetent yeast by modifying previously described methods (60). We selected the yeast transformations by plating on SD -HIS/-URA 2% agar plates. We scraped the yeast transformations off the selective plates using cell spreaders, resuspended in SD -HIS/-URA and froze them in 15% glycerol stocks at −80°C for subsequent experiments. We generated 19 million transformants, with an average representation of 963 cells per guide/donor oligonucleotide.

### Pooled editing and plasmid curing

We carried out pooled editing for CRISPEY-BAR+ similarly to previous work (24, 27, 28). We thawed the yeast library from frozen glycerol stocks and added to 200 mL SD -histidine -uracil 2% raffinose, starting at OD_600_ = 0.4, and incubated at 30°C shaking at 250 RPM for 24 h. After this phase of growth in raffinose we induced editing with galactose. To begin this new phase, we re-inoculated the pre-editing cultures grown in raffinose in 200 mL SD -uracil 2% galactose, with a starting OD_600_ = 0.4, and incubated at 30°C shaking at 250 RPM for 24 h. This phase of editing induced by galactose lasted for 72 h. We transferred the yeast library to 200 mL of fresh SD -histidine -uracil 2% galactose every 24 h. We harvested the cells from the last growth in galactose and stored them in SD - histidine -uracil 2% glucose with 15% glycerol at −80°C.

We then removed CRISPEY-BAR+ plasmids after editing, by growing them in 200 mL of SD 2% glucose media starting at OD_600_ = 0.4 and incubated at 30°C shaking at 250 RPM for 24 h to remove plasmid selection. After this phase of non-selective growth, were-incolulated to 200 mL SD 2% glucose with 1g/L of 5-fluoroorotic acid monohydrate (5-FOA) (GoldBio) starting at OD_600_ = 0.4 and incubated at 30°C shaking at 250 RPM for 24 h to select against the *URA*-bearing CRISPEY-BAR+ plasmid (61). We froze the edited, plasmid-free yeast library and stored it in 15% glycerol stocks at −80°C in preparation for the growth competitions.

### Pooled growth competitions

We carried out pooled competitions in 500 mL DeLong flasks (with a flat bottom) in 100 mL of M3 -uracil -lysine -leucine media. We thawed edited and plasmid-cured yeast cells (see Pooled editing and plasmid curing) in 15 mL of SC media at 30°C shaking at 250 RPM overnight in a 500 mL flask. We inoculated 100 mL M3 -uracil -lysine -leucine media starting at OD_600_ = 0.4 and grew at 30°C shaking at 250 RPM for 24h. We then inoculated 500 μL of this saturated culture into 100 mL of fresh media in 500 mL DeLong flasks. This culture was then grown at 30°C in an incubator sharing at 250 RPM with incubation time varying depending on the experimental condition: 24 h (1-Day), 48 h (2-Day), 72 h (3-Day), or 120 h (5-Day). We included three replicates per condition. After incubation, we transferred 500 μL of the saturated culture into fresh media in a new flask. This serial dilution was continued 6 times, yielding 6 time-points over which to measure the rate at which a barcode frequency changed. We pre-warmed fresh media before each transfer. Contamination caused the loss of the last three time-points for one of the three replicates of the 2-Day condition. After each transfer of 500 μL, we froze the leftover 9500 μL so that we could later sequence the barcodes present at each time-point. To prepare this culture for freezing, we transferred it to 50 mL conical tubes, spun down at 3000 RPM for 5 min, resuspended in glycerol15%, aliquoted into three 1.5 mL tubes, and stored at −80°C.

### DNA extractions

After completing the growth competitions, we extracted DNA from frozen samples following a modified protocol that improves the yield of extraction (56). For each sample, a single tube of the three that were frozen for each sample (see Pooled growth competitions) was removed from the freezer and thawed at room temperature. We extracted DNA from 400 μL of that sample, and stored the remaining culture at −80°C, using the following modification of the Lucigen MasterPure yeast DNA purification kit (#MPY80200). We transferred the thawed cells into a 2 mL tube and centrifuged at 3500 RPM for 3 min. After discarding the supernatant, the pellet was then resuspended in 400 μL of sterile water, spun again at 3500 RPM for 3 min. After discarding the supernatant, the pellet was resuspended with 1200 uL of the MasterPure lysis buffer supplemented with 8 μL of zymolyase and 4 μL of RNase A (ThermoFisher). We split the mix into two 1.5 mL centrifuge tubes which we incubated at 37°C for 1 h. After such incubation, we added 2 μL of zymolyase to each tube, and then we incubated them at 65°C for 4 h, vortexing the tubes every hour. We then put the tubes on ice for 10 min and then we mixed 300 μL of MPC Protein Reagent with the solution of each tube by vortexing. We separated protein and cell debris by centrifugation at 10,000 RPM for 15 min, transferring 800 μL of supernatant from each tube to a 2 mL centrifuge tube. We further separated remaining protein and cell debris by centrifuging at 13200 RPM for 5 min. Next, we added 500 μL of isopropanol to each tube, mixed by inversion, centrifuged at 10,000 RPM for 15 min, and discarded the supernatant. We washed the pellet by adding 500 μL of ethanol 70%, mixing by inversion, and then centrifuging the tubes at 10,000 RPM for 7 min. We discarded the supernatant and the tubes were left to air dry. We resuspended the pellet, containing the DNA, in 50 μL of Elution Buffer. Next we combined the two tubes per sample into a single 1.5 mL DNA LoBind^®^ tube. We added 2 μL of 5 ng/μL RNAse A and incubated at 37°C for 30 min for further digestion and quantified the amount of DNA by Qubit dsDNA BR assay (Invitrogen).

### Sequencing library preparation

We amplified 10 μg of genomic DNA in a 400 uL Q5 polymerase (New England Biolabs) reaction with 1uM forward primer #261 and 1 μM reverse primer equimolar mix of primers #327-#334. We performed the PCR reaction with 1M betaine and initial denaturation of 98°C for 2 min, then 18 cycles of 98°C for 10 s, 65°C for 20 s, then extension at 70°C for 5 min. The staggered primers provide additional sequence complexity for read2 during the sequencing of the barcode. We purified 100 μL of this first-step PCR with 100 μL of magnetic beads (NucleoMag NGS Clean-up and Size Select, REF 744970.5). The purified amplicons were indexed by 50 μL Q5 polymerase (New England Biolabs) reaction with 1 μM equimolar mix of indexing primers for Illumina sequencing. The PCR thermocycling conditions were as follows: initial denaturation of 98°C for 2 min, then 8 cycles of 98°C for 10 s, 70°C for 20 s, then extension at 70°C for 5 min. We purified the indexed amplicons with 50 μL beads and eluted them in 100 uL of water. We quantified them using Qubit dsDNA HS (Invitrogen). We mixed in equimolarity the purified, indexed amplicons from six time point samples for each of the three replicates per condition and purified them using a SizeSelect II gel (Invitrogen) for a ~300 bp product. We then purified the size-selected libraries with beads. We submitted the libraries to the CZ Biohub SF for paired-end sequencing in a flow cell (4 lanes) of NovaSeq S4 using custom read1 primer #354.

### Estimating edited variant effect on fitness

Incorporating multiple barcodes per edit enhances the precision of fitness measurements by increasing biological replication within a flask. This approach enables the detection of random mutants that may arise during transformation, editing, or competition as well as cases where the specific edit failed in the CRISPEY-BAR+ procedure as these appear as outliers, thereby improving the accuracy of our estimates of variant fitness effects. Spontaneous mutations with strong positive fitness effects, in particular, can disproportionately dominate the reads for a given variant. However, these mutations are unlikely to impact multiple independently edited barcodes. To mitigate false positives, we removed these outlier barcodes by discarding the barcode with the highest read count for each variant.

To infer fitness from the barcode count trajectories, we followed a variational Bayesian approach (44). Briefly, our objective is to infer the fitness values $\vec{s}=(s^{\left(1\right)},s^{\left(2\right)},\ldots ,s^{\left(N\right)})$ for all N strains. For a specific strain j, our data consists of a vector of barcode counts across all time points sequenced in the experiment,

$$r^{\left(\vec{j}\right)}=(r_{1}^{\left(j\right)},r_{2}^{\left(j\right)},\ldots ,r_{T}^{\left(j\right)}),$$

for the T time points in our experiment. The entire dataset then consists of N vectors of raw reads

$$\underline{R}=(r^{\left(\vec{1}\right)},r^{\left(\vec{2}\right)},\ldots ,r^{\left(\vec{N}\right)}).$$

Our fitness models for how the allele frequency of barcode j changes over time takes the form

$$f_{t+1}^{\left(j\right)}=f_{t}^{\left(j\right)}exp\;((s^{\left(j\right)}-\overset{-}{s_{t}})\tau ),$$

where $f_{t}^{\left(j\right)}$ is the allele frequency of barcode j at time $t,\;{\overset{‾}{s}}_{t}$ is the population mean fitness at time t, and τ is the time interval between t and t+1. Thus, we must include the inference of the nuisance parameter ${\overset{‾}{s}}_{t}$ for each time point in our experiment. To constrain the value of this parameter, the experiment includes a series of what we consider to be neutral barcodes (barcodes associated with non-editing oligos) whose fitness is zero by definition. This means that we include a series of barcodes that, by definition, have

$$s^{\left(n\right)}=0.$$

Furthermore, the barcode frequency $f_{t}^{\left(j\right)}$ is not what our experiments quantify. Naively, we could simply assume that

$$f_{t}^{\left(j\right)}=\frac{r_{t}^{\left(j\right)}}{\sum_{k=1}^{N}r_{t}^{\left(k\right)}},$$

but this would assume we have perfect readouts for the experiment. Since we use a Bayesian framework, we can simply include the computation of all frequencies

$$\underline{F}=(f^{\left(\vec{1}\right)},f^{\left(\vec{2}\right)},\ldots ,f^{\left(\vec{N}\right)}),$$

as nuisance parameters in our inference.

By Bayes theorem, our inference problem can be expressed as

$$\pi (\vec{s},\vec{\overset{‾}{s}},\overset{‾}{F}|\underline{R})\propto \pi (\underline{R}|\vec{s},\vec{\overset{‾}{s}},\vec{F})\pi (\vec{s},\vec{\overset{‾}{s}},\overset{‾}{F}),$$

where $\vec{\overset{‾}{s}}=(\overset{-}{s_{1}},\overset{-}{s_{2}},\ldots ,\overset{-}{s_{T}})$, and π(⋅) is a probability distribution. Although this is an intractable problem, Razo-Mejia *et al.* (44) deploys a variational approach to solve this inverse problem of extracting fitness values from raw read counts.

Furthermore, using a hierarchical Bayesian model, the Bayesian framework can be extended to combine the information from multiple experimental replicates into a single inference problem. Hierarchical models systematically account for all available information and propagate sources of uncertainty throughout the inference process, increasing the statistical power when inferring the fitness values. The results shown in the main text take this hierarchical approach as follows: The reads for all individual barcodes mapping to a specific edit were aggregated to be used as a single number $r_{t}^{\left(j,n\right)}$ for edit j and replicate n at time t. We then assume that we can infer the corresponding fitness $s^{\left(j,n\right)}$ as a sample from a hyper-parameter distribution (the hyperfitness) $\phi^{\left(j\right)}$ shared among all edits j across all replicates. The objective then becomes inferring the values for all hyperfitness values $\vec{\phi }=(\phi^{\left(1\right)},\phi^{\left(2\right)},\ldots ,\phi^{\left(E\right)})$ for all E edits and the individual replicate fitness values $\overset{‾}{S}=(s^{\left(\vec{1}\right)},s^{\left(\vec{2}\right)},\ldots ,s^{\left(\vec{M}\right)})$ for all M experimental replicates given the corresponding raw reads $\vec{R}=(r^{\left(\vec{1}\right)},\ldots ,r^{\left(\vec{M}\right)})$. This inference problem can be written by Bayes theorem as (we ignore the corresponding nuisance parameters for simplicity although they are included in the full inference; see (44) for more details)

$$\pi (\vec{\phi },\overset{‾}{S}|\underline{R})\propto \pi (R|\overset{‾}{S})\pi (\overset{‾}{S}|\vec{\phi })\pi (\vec{\phi }).$$

In this way, all experiments are connected via the hyperfitness parameter, bringing all information from each replicate and the corresponding uncertainty. The main text results are based on the posterior distributions of the hyperfitness parameters.

### Variant properties

We annotated variants using Variant Effect Predictor (VEP) (62). We retrieved the predicted protein structures of all *S. cerevisiae* S288C genes in Supplementary Table 1 from the AlphaFold Protein Structure Database (63, 64). There are no protein structures for *IRA1*, *IRA2*, and *SDC25*. We measured solvent accessibility using DSSP (65, 66). We measured packing density as the number of other ɑ-carbons within 10Å of the ɑ-carbon for a given residue using custom code. We used FoldX to determine the destabilizing effect of nonsynonymous variants (49). We retrieved SIFT scores from the mutfunc database (67). We obtained the allele frequency and heterozygosity of each variant from the 1,011 yeast genomes collection (17). From the same source we obtained the number of singleton variants for each of the 1,011 strains and their ploidy.

### Matching non-neutral variants to neutral controls

When comparing the distribution of non-neutral and neutral variants in natural populations of *S. cerevisiae* we need to account for the fact that non-neutral variants have a different frequency distribution to neutral variants (Fig. 3A). To that end, for each non-neutral variant we first identified all unmatched neutral variants with the smallest absolute difference in frequency in the 1,011 natural isolates. When multiple candidate neutral variants were available, we filtered to the variant with the minimal Jaccard similarity to the non-neutral variant to avoid pairing linked variants with identical phylogenetic distributions (see Variant co-occurrence analysis). In cases where multiple candidates were still present, a non-neutral variant was selected at random. After running this procedure, we confirmed that the set of non-neutral variants had near identical allele-frequency to their matched neutral control variant (Fig. S1C). We also confirmed that matched pairs only co-occur at high allele frequency in line with expected co-occurrence if variant pairs were sampled at random (dotted line in Fig. S1D). For other variants of interest (such as variants beneficial in at least one environment) we adopted the same procedure to compare variants of interest to variants outside of the set.

### Constructing a maximum likelihood phylogenetic tree

To construct a maximum likelihood phylogenetic tree for the 1,011 natural isolates we used the published whole-genome sequencing data from Peters *et al.* (17). We started with the previously published VCF file which contains every single-nucleotide polymorphism (SNP) and indel that has been called at the population level (1011Matrix.gcvf). We filtered for SNP sites present in all strains and where the minor allele could be confidently called in at least 2 strains and could thus be parsimony-informative. A maximum likelihood tree was generated based on the 866,130 filtered SNPs using IQTREE2 (iqtree2 -s filtered_snps.min4.phy -m GTR+ASC) (68). The resulting maximum likelihood tree was rooted using the Taiwanese clade as an outgroup as this represented the most divergent population (17). This rooted tree was used in all subsequent analyses (Fig. 1C).

For each of the 9,447 variants for which we have barcodes in the competition data we characterized its phylogenetic distribution using several statistics commonly used to study the distribution and evolution of categorical traits. Specifically for every non-singleton variant we calculated the parsimony and retention index as well as its phylogenetic signal (69). We also performed ancestral state reconstruction using the ace function in R (type = “discrete”, method = “ML”, model = “ER”). From the resulting ancestral state estimates we quantified the rate of evolution (transition rate), number of independent origins of each variant along a branch (0–1 transitions) and relative allele age (ancestral node depth / tree depth). We found no statistically significant difference for any of these 6 statistics between non-neutral and the matched set of neutral control variants, suggesting that the phylogenetic distributions of neutral and non-neutral variants do not differ in any systematic manner.

### Variant co-occurrence analysis

For each pair of alleles (i and j) the probability of co-occurring in the same natural isolate was calculated as the Jaccard similarity coefficient:

$$J(A,B)=\frac{A⋂B}{A⋃B}$$,

where A is the set of natural isolates in which allele i is present and B Is the set of natural isolates in which allele j is present. Using this measure, we quantified whether pairs of non-neutral variants where significantly less likely to co-occur than pairs of neutral variants as this would suggest that they were under purifying selection (since a deleterious variant is likely to go extinct before a second deleterious variant can arise in the same genetic background). For singletons we calculated the mean Jaccard similarity for all pairs of singleton non-neutral variants and then compared this with the mean Jaccard similarity for all pairs of neutral singleton variants (Fig. S1A). For non-singletons we calculated the mean Jaccard similarity of non-neutral variants and compared this to the mean Jaccard similarity for all pairs of frequency-matched neutral control variants (Fig. S1B). To determine whether the difference in means were statistically significant we repeated this calculation for 1,000 bootstrap replicates (*p*-values in Fig. S1A,B).

### Quantifying the enrichment of beneficial variants in domesticated clades

In each of the 1,011 natural isolates we quantified the number of variants that were beneficial in at least one environment (*N*_beneficial_) and compared to this the number of matched control variants (*N*_comtrol_). As we are comparing the same number of beneficial variants to matched controls variants and since these two sets of variants have near-identical frequency distribution in the natural isolates, each natural isolates is expected to contain an equal number of beneficial and matched controls variants (i.e, *N*_beneficial_ - *N*_control_ = 0). A strain was considered to be enriched for beneficial variants if *N*_beneficial_ - *N*_control_ was significantly larger than 0 (Fig. 4C).

The majority of 1,011 natural isolates were identified in Peters *et al.* (17) as belonging to one of 26 distinct clades (813 strains in total). Of these 26 clades, 17 “clean” clades were studied by Tengölics *et al.* (45). In Tengölics *et al.*, 10 clades are assigned as “domesticated” (600 strains) whereas 7 are assigned as “wild” (56 strains). Strains from all other clades and mosaic clades were assigned as “other” (355 strains) in our analysis (Fig. S3). Each of these 3 groups includes strains isolated from a variety of different environments and ecologies and we classified the ecological origins as either “industrial,” “natural” or “other.” Specifically, 587 strains were classified as “industrial” if their ecological origin was listed as either beer, bakery, industrial, wine, distillery, palm wine, bioethanol, fermentation, cider, dairy or sake. 392 strains were classified as “natural” if their ecological origin was listed as either water, fruit, flower, human-clinical, insect, nature, tree, soil or human. Finally, 32 strains were classified as “other” if their ecological origin was listed as either lab strains, probiotic or unknown. The classification of the 1,011 strains as belonging to “domesticated,” “wild,” or “other” clades, and their ecological origins (industrial, natural, or other) can be observed in the maximum likelihood tree in Fig. 1C.

We first determined whether domesticated strains were significantly enriched for beneficial or non-neutral variants when compared to wild strains (Wilcoxon test, *p*-value < 0.05, Fig. 4C). We then repeated this comparison iteratively removing variants above a threshold allele frequency for all allele frequencies observed in the 1,011 natural isolates (Fig. 4D). We repeated this analysis comparing industrial and natural strains within the wild and domesticated populations (Fig. S2B).

We used a linear mixed-effects model to test whether strains isolated from industrial environments (e.g., wine or beer) were enriched for beneficial variants (higher *N*_beneficial_ – *N*_control_) compared to strains in the same clade isolated from natural environments (e.g., flowers or insects) (17). Specifically, we fit a mixed-effects model using the *lmer* function from the *lme4* package in *R*, with the ecological origin of the strains (industrial vs. natural) as a fixed effect and strain clade identity as a random intercept (model formula: enrichment ~ ecology + (1 | clade)). In Figure S3, we report the fixed effect estimate of being isolated from an industrial environment compared to a natural environment, along with the corresponding *p*-value, which we estimated using the Satterthwaite approximation via the *lmerTest* package in *R*.

## Supplementary Material

Supplement 1
