# Supplementary material for: Massively parallel experimental interrogation of natural variants in ancient signaling pathways reveals both purifying selection and local adaptation
Source: bioRxiv. 2024 Oct 31:2024.10.30.621178. Preprint. [Version 1] doi: 10.1101/2024.10.30.621178 (PMC11565963; doi:10.1101/2024.10.30.621178)
Supplement: Supplement 1 [file NIHPP2024.10.30.621178v1-supplement-1.pdf]

bioRxiv preprint doi: https://doi.org/10.1101/2024.10.30.621178; this version posted October 31, 2024. The copyright holder for this preprint (which was not certified by peer review) is the author/funder. This article is a US Government work. It is not subject to copyright under 17 USC 105 and is also made available for use under a CC0 license.

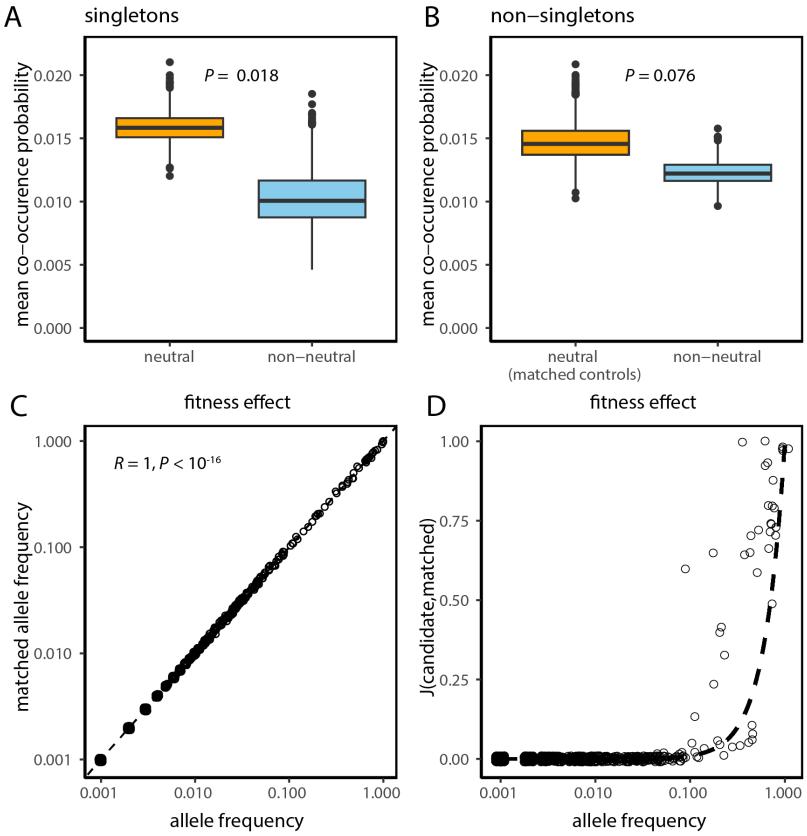

Figure S1. **To accompany Figure 3. Non-neutral variants show evidence of negative selection.** (A) Box plot showing the co-occurrence probability for neutral and non-neutral singleton variants. Data shown are the mean co-occurrence probability for 1,000 bootstrap samples (Methods). To compare non-singleton non-neutrals against non-singleton neutrals, we matched each non-neutral variant to a neutral variant with comparable frequency. These matched controls ensure that our calculation of the frequency of co-occurrence is not confounded by the fact that non-neutrals tend to have lower allele frequencies than neutral variants (Fig. 3A). (B) Box plot showing the co-occurrence probability for neutral matched controls and non-neutral non-singleton variants. Data shown are the mean co-occurrence probability for 1,000 bootstrap samples. Boxplots show the median and upper and lower quartiles; whiskers show 1.5 times the interquartile range. The *p*-values for the group comparisons are the fraction of bootstrap samples in which mean co-occurrence probability for neutrals is lower than for non-neutral. (C) The relationship between the allele frequency of non-neutral variants (*x*-axis) and the allele

bioRxiv preprint doi: https://doi.org/10.1101/2024.10.30.621178; this version posted October 31, 2024. The copyright holder for this preprint (which was not certified by peer review) is the author/funder. This article is a US Government work. It is not subject to copyright under 17 USC 105 and is also made available for use under a CC0 license.

frequencies of their matched neutral controls (*y*-axis). We report Pearson's correlation coefficient and corresponding *p*-value. (D) The relationship between allele frequency and Jaccard similarity coefficient for the set of strains carrying a non-neutral variant and the set of strains carrying the matched control variant.

bioRxiv preprint doi: https://doi.org/10.1101/2024.10.30.621178; this version posted October 31, 2024. The copyright holder for this preprint (which was not certified by peer review) is the author/funder. This article is a US Government work. It is not subject to copyright under 17 USC 105 and is also made available for use under a CC0 license.

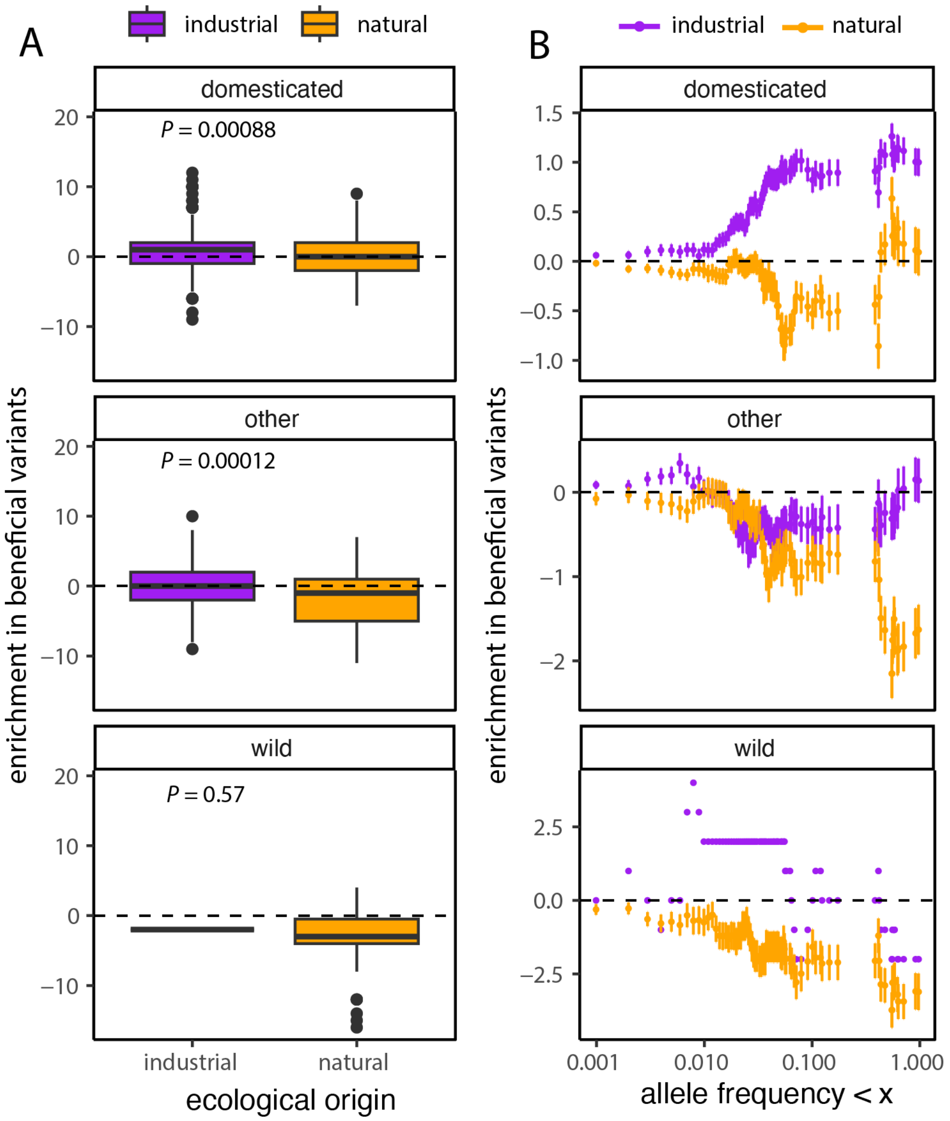

Figure S2. To accompany Figure 4. Common non-neutral variants show evidence of local adaptation. (A) Boxplot showing the difference in enrichment in variants that are beneficial in at least one environment between strains from industrial or natural sources within the clades classified as domesticated, wild, or other in Fig. 1C. The *p*-values for the group comparisons were calculated using the Wilcoxon's test. Boxplots show the median and upper and lower quartiles; whiskers show 1.5 times the interquartile range. (B) The enrichment in beneficial variants as a function of allele frequency for strains from industrial, natural, or other sources within the clades classified as domesticated, wild, or other in Fig. 1C.

bioRxiv preprint doi: https://doi.org/10.1101/2024.10.30.621178; this version posted October 31, 2024. The copyright holder for this preprint (which was not certified by peer review) is the author/funder. This article is a US Government work. It is not subject to copyright under 17 USC 105 and is also made available for use under a CC0 license.

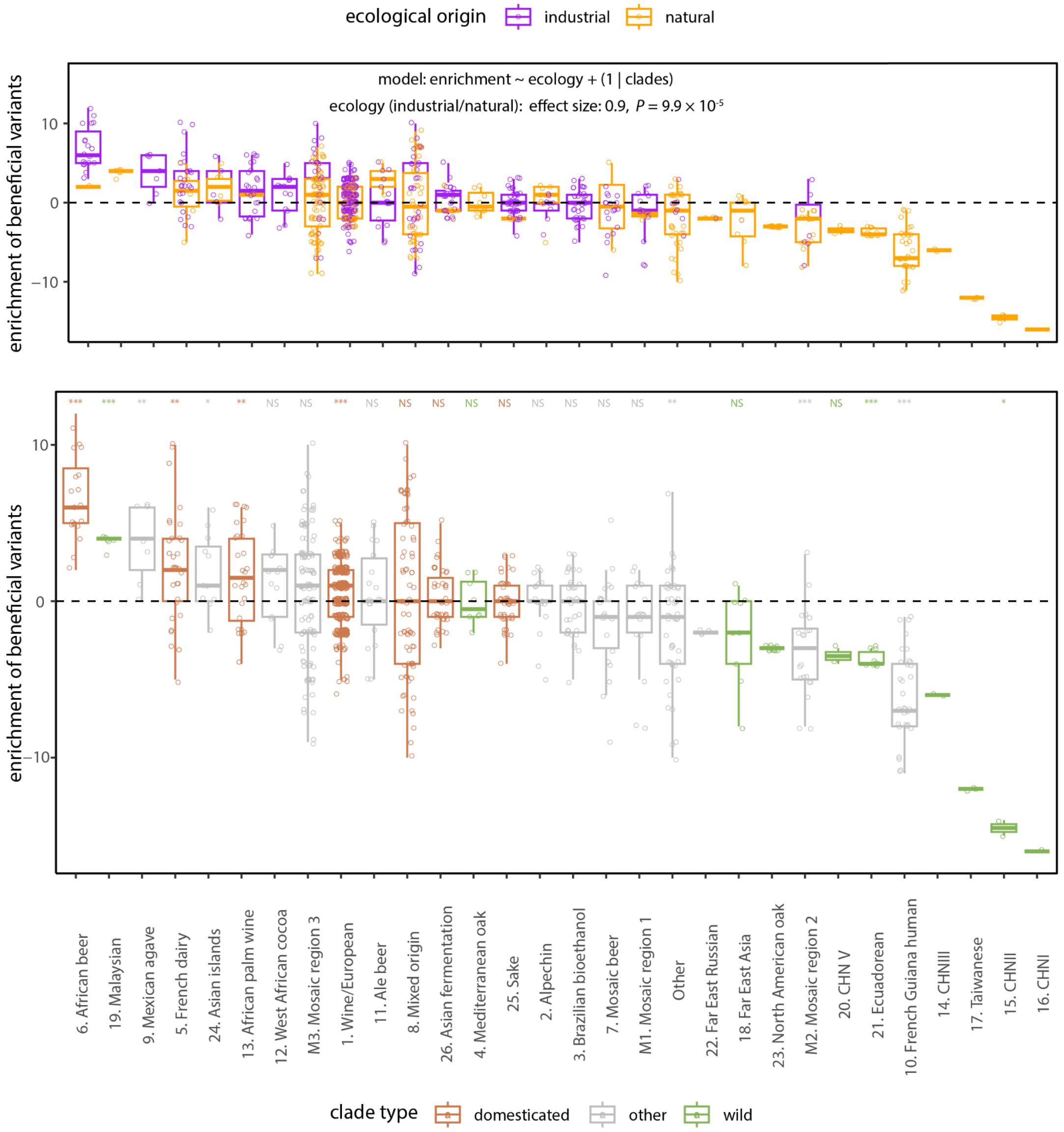

Figure S3. To accompany Figure 4. Common non-neutral variants show evidence of local adaptation. Boxplots show the enrichment in beneficial variants in at least one condition for all *Saccharomyces cerevisiae* clades. Clades are arranged from highest to lowest enrichment. The significance levels for each clade are coming from *t*-test to determine if there are differences in terms of enrichment between industrial and natural strains (*p*-value = 0.001: ***, *p*-value = 0.01: *, *p*-value = 0.05: **, *p*-value > 0.05: NS). We fit a linear mixed-effects model to quantify the impact of ecology while treating clade identity as a random effect. Boxplots show the median and upper and lower quartiles; whiskers show 1.5 times the interquartile range.

bioRxiv preprint doi: https://doi.org/10.1101/2024.10.30.621178; this version posted October 31, 2024. The copyright holder for this preprint (which was not certified by peer review) is the author/funder. This article is a US Government work. It is not subject to copyright under 17 USC 105 and is also made available for use under a CC0 license.

Supplementary Table S1: Genes considered in this study

Standard name	Systematic name	Pathway
BCY1	YIL033C	Ras/PKA
CDC25	YLR310C	Ras/PKA
CYR1	YJL005W	Ras/PKA
GPA2	YER020W	Ras/PKA
GPB1	YOR371C	Ras/PKA
GPB2	YAL056W	Ras/PKA
GPR1	YDL035C	Ras/PKA
IRA1	YBR140C	Ras/PKA
IRA2	YOL081W	Ras/PKA
PDE1	YGL248W	Ras/PKA
PDE2	YOR360C	Ras/PKA
RAS1	YOR101W	Ras/PKA
RAS2	YNL098C	Ras/PKA
RGS2	YOR107W	Ras/PKA
TFS1	YLR178C	Ras/PKA
TPK1	YJL164C	Ras/PKA
TPK2	YPL203W	Ras/PKA
TPK3	YKL166C	Ras/PKA
KOG1	YHR186C	TOR/Sch9
LST8	YNL006W	TOR/Sch9
SCH9	YHR205W	TOR/Sch9
TCO89	YPL180W	TOR/Sch9
TOR1	YJR066W	TOR/Sch9
TOR2	YKL203C	TOR/Sch9
